# Enhancing gene set overrepresentation analysis with large language models

**DOI:** 10.1093/bioadv/vbaf054

**Published:** 2025-03-13

**Authors:** Jiqing Zhu, Rebecca Y Wang, Xiaoting Wang, Ricardo Azevedo, Alexander Moreno, Julia A Kuhn, Zia Khan

**Affiliations:** Alector, Inc, South San Francisco, CA 94080, United States; Alector, Inc, South San Francisco, CA 94080, United States; Alector, Inc, South San Francisco, CA 94080, United States; Alector, Inc, South San Francisco, CA 94080, United States; Alector, Inc, South San Francisco, CA 94080, United States; Alector, Inc, South San Francisco, CA 94080, United States; Alector, Inc, South San Francisco, CA 94080, United States

## Abstract

**Motivation:**

Gene set overrepresentation analysis (ORA) is widely used to interpret high-throughput transcriptomics and proteomics data, but traditional methods rely on human-curated gene set databases that lack flexibility.

**Results:**

We introduce *llm2geneset*, a framework that leverages large language models (LLMs) to dynamically generate gene set databases tailored to input query genes, such as differentially expressed genes and a biological context specified in natural language. These databases integrate with methods, such as ORA, to assign biological functions to input genes. Benchmarking against human-curated gene sets demonstrates that LLMs generate gene sets comparable in quality to those curated by humans. *llm2geneset* also identifies biological processes represented by input gene sets, outperforming traditional ORA and direct LLM prompting. Applying the framework to RNA-seq data from iPSC-derived microglia treated with a *TREM2* agonist highlights its potential for flexible, context-aware gene set generation and improved interpretation of high-throughput biological data.

**Availability and implementation:**

*llm2geneset* is available as open source at https://github.com/Alector-BIO/llm2geneset and via a web interface at https://llm2geneset.streamlit.app.

## 1 Introduction

Gene sets, which group genes or proteins based on shared biological functions, pathways, or regulatory mechanisms, are essential tools in transcriptomics and proteomics for interpreting complex datasets (Maleki *et al.* 2020, Mubeen *et al.* 2022). By focusing on coordinated biological processes rather than individual genes, gene set analysis enhances the understanding of underlying biology and helps identify patterns that may be obscured in high-dimensional data. This approach allows molecular changes, such as differentially expressed genes (DEGs), to be connected to specific biological processes, supporting investigations into normal physiology, disease mechanisms, and therapeutic strategies. The generation and interpretation of gene sets are critical for deriving meaningful insights from transcriptomics and proteomics studies and for deepening our understanding of biological systems.

Methods for gene set enrichment analysis, such as overrepresentation analysis (ORA), are widely used to evaluate whether gene sets are significantly associated with a list of DEGs (Subramanian *et al.* 2005, Huang *et al.* 2009, Wu *et al.* 2010, Wu and Smyth 2012, Kuleshov *et al.* 2016). These methods typically rely on external, human-curated gene set databases, which must be regularly updated to incorporate advances in scientific knowledge. However, these databases often pose challenges when tailoring gene sets to specific research questions or experimental contexts, and the choice of gene sets can strongly influence the interpretation of transcriptomics and proteomics studies (Mubeen *et al.* 2022). In this paper, we demonstrate how large language models (LLMs) enable the generation of gene sets using natural language. This approach supports the customization of gene sets for specific research questions and enhances the identification of biologically relevant processes when integrated with enrichment analysis.

LLMs have recently found several uses in the analysis of transcriptomics datasets and their application to problems in computational biology is an active area of research. LLMs have been used to automate cell type annotation in single-cell datasets on the basis of their ability to capture marker genes of known cell types (Hou and Ji 2023). Recent work has evaluated whether LLMs can discern functional relationships within a given set of genes by prompting LLMs (Joachimiak *et al.* 2023, Hu *et al.* 2025). LLMs have been augmented with bioinformatic specific tools to solve tasks (Jin *et al.* 2024). Combining these approaches, LLMs as agents have been explored to discern the function of an input set of genes using self-verification approaches (Wang *et al.* 2024). Yet, use and evaluation of LLMs as a tool for flexible gene set generation and overrepresentation analyses has not been explored.

**Figure 1.**
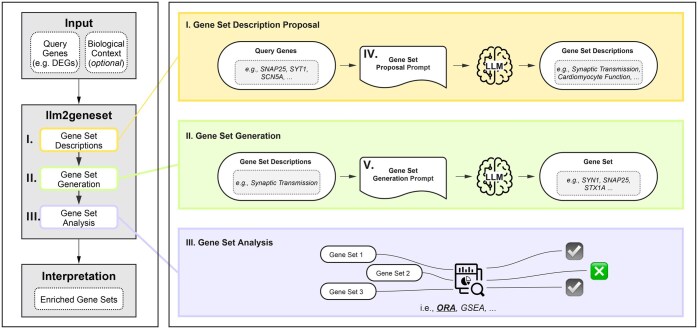
Overview of the *llm2geneset* framework. The framework takes a set of query genes [e.g., differentially expressed genes (DEGs)] and an optional biological context as input. The LLM-based gene set database construction involves two stages: first, proposing biological processes relevant to the query genes within the context, and second, generating gene sets by associating relevant genes with these processes. The resulting customized gene set database can be utilized by gene set analysis methods, such as overrepresentation analysis (ORA), to identify enriched gene sets.

In this study, we introduce *llm2geneset*, a framework that leverages LLMs to generate a gene set database tailored to input query genes (e.g. DEGs) and a biological context specified in natural language (Fig. 1). This dynamically generated database can be used by gene set analysis methods, such as ORA, to assign biological processes and functions to the input genes. We evaluate two key aspects of the framework: its ability to accurately generate gene sets from natural language descriptions and its ability to map input gene sets to known functions. Using human-curated gene sets, we test whether LLMs can recover genes curated by humans when provided with a natural language description and find that they perform comparably to human curators, with prompting strategies tuning precision. Additionally, we evaluate whether *llm2geneset* can identify the biological processes or functions represented in input gene sets, whether corresponding to single or multiple processes, and find that it outperforms traditional ORA and direct LLM prompting. Finally, we demonstrate the framework’s potential as a flexible and steerable tool for gene set analysis by applying it to transcriptomics data from iPSC-derived microglia treated with a *TREM2* agonist.

## 2 Methods

Detailed methods are provided in the Supplementary material. We used the following versions of OpenAI LLMs: GPT-4o mini (gpt-4o-mini-2024–07-18), GPT-3.5 (gpt-3.5-turbo-0125), and GPT-4o (gpt-4o-2024–08-06).

We used the following prompt to generate gene sets:

List all the known genes directly and indirectly involved in the following biological process or cellular component “““{d e scr}”””. Use the following JSON schema:

′ ′ ′json

{{

“type”: “array”,

“items”: {{

“type”: “object”,

“properties”: {{

“gene”: {{

“type”: “string”,

}}

}},

“required”: [“gene”]

}}

}}

′ ′ ′

The field “gene” is a gene involved in the following biological process or cellular component: “““{descr}”””. Use the HUGO Gene Nomenclature Committee (HGNC) gene abbreviations. Place the output in a JSON code block. Do not add any comments in the JSON code block.

In the above prompt {descr} was replaced with a natural language description of a gene set. The above prompt, query to an LLM, and parsing can be concisely written as a function that takes as input a string description of a gene set and returns a string list with the corresponding gene symbols:

G:List[str] <- GetGenes(D:str)

We used the following prompt to propose a set of gene set descriptions from a list of genes.

List {n_pathways} biological pathways, biological processes, or cellular components that contain the following genes “““{genes}””” with high confidence. Be as specific as possible. List non-overlapping pathways, processes, or components. Do not i n clude the gene names in the outputs. Use the following JSON schema:

′ ′ ′json

{{

“type”: “array”,

“items”: {{

“type”: “object”,

“properties”: {{

   “p”: {{

“type”: “string”,

}},

}},

“required”: [“p”]

}}

}}

′ ′ ′

Example output will look like the following:

′ ′ ′json

[{{“p”:”BP or Pathway 1”}},

{{“p”:”BP or Pathway 2”}},

{{“p”:”BP or Pathway 3”}},

{{“p”:”BP or Pathway 4”}}

′ ′ ′

The element “p” designates a pathway, biological process or cellular component. Place the output in a JSON code block. Do not add any comments in the JSON code block.

In the above prompt, {n_pathways} was replaced with the number of desired biological pathways and process descriptions. {genes} was replaced by a comma-separated list of genes for which we requested these biological processes and pathways. As with the gene set generation prompt, we used the https://pypi.org/project/json-repair/ package to parse out the returned genes and repair any minor JSON formatting errors. If the JSON output was not repairable, we queried the model again with a different seed value. To steer the model toward experimentally relevant biological processes and pathways, we additionally modified the above prompt to include contextual information when this contextual information was provided:

List {n_pathways} biological pathways, biological processes, or cellular components that contain the following genes “““{genes}””” with high confidence. Also consider the following context as related to the genes: “““{context}””” when selecting pathways, processes, and components.

Here, {context} was replaced with a user-provided string that provided additional context to steer the model generations (e.g. “*in vitro* microglia treated with a *TREM2* agonist antibody”). The above can be concisely written as a function that takes as input a list of genes D, a requested number of processes and pathways N, and optionally a C context string:

P:List[str] <- GetPathwaysProcesses(D:List[str], N:int, C:str)

*llm2geneset* proposes pathways and biological process descriptions based on the input set of genes and an experimental context. These pathway descriptions, alone, are used to generate gene sets which are then tested for overrepresentation in the input set of DEGs. The GetGenes() function call below does not have access to any previous context. The parameters of the algorithm are as follows:

- D = set of DEGs (or any set of genes)
- N = number of biological pathways and processes to propose
- C = (optional) contextual information regarding the experiment from which the DEGs were obtained
- B = number of background gene sets for ORA

Pseudo-code is provided below:

llm2geneset(D: List[str], N: int, C: str)

R = []

P = GetPathwaysProcesses(D)

for pathway in P:

G = GetGenes(pathway)

p = hgsf(|intersect(D, P)|- 1, B, |G|, |D|)

    R  =  R.append((pathway, p))

  return padjust(R)

In the above pseudo-code, the function padjust() computes *q*-values to account for multiple testing. hgsf() is one minus the cumulative distribution function (1−CDF) of the hypergeometric distribution. The function call computes the *P*-value according to a one-tailed Fisher’s exact test. *llm2geneset* returns a list of N pathways sorted on their overrepresentation *P*-values.

## 3 Results

### 3.1 LLMs generate informative gene sets

We evaluated whether LLMs could be used to generate gene sets using flexible natural language descriptions of these gene sets. To achieve this, we used a fixed text prompt with a field that can be replaced by the natural language description of the gene set (see Section 2) (Sahoo *et al.* 2024, Schulhoff *et al.* 2024). Our prompt consisted of several elements: (i) a role-prompt, a strategy whereby a system message is used to guide the generations of an LLM; (ii) the prompt itself requesting generation of genes for a given biological process or cellular component; and (iii) a formatting component to guide the LLM to produce output using standard HGNC gene symbols that can be programmatically loaded into data structures for downstream use in gene set tests (see Section 2).

**Figure 2.**
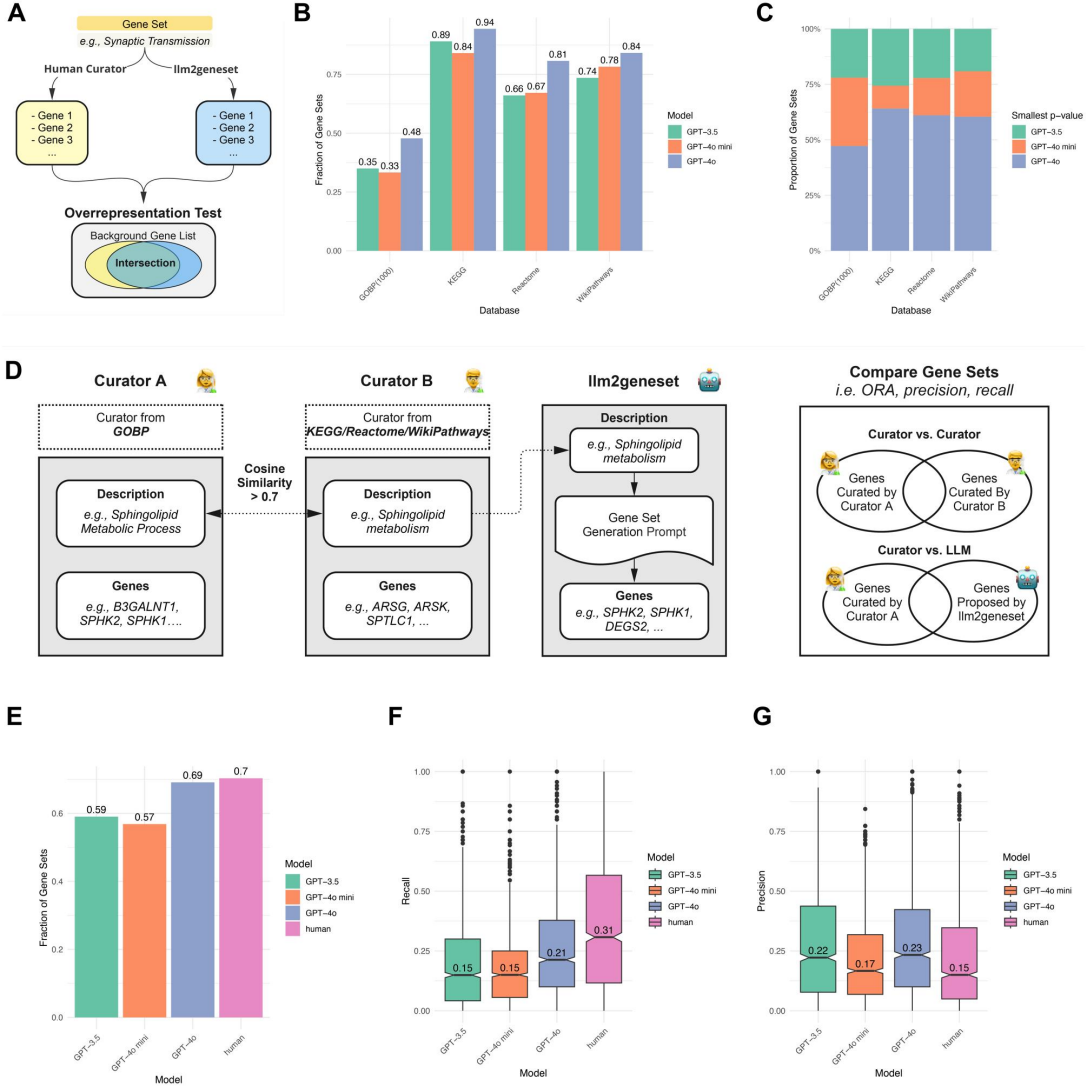
Evaluating gene sets generated by LLMs. (A) Gene sets generated by LLMs were assessed for significant overrepresentation in human-curated gene sets. Significant overrepresentation indicates that the LLM accurately associates gene set descriptions with corresponding genes. (B) Fraction of gene sets showing significant enrichment in curated sets at a Bonferroni-adjusted *P*-value of .01 across databases and LLMs. Gene Ontology Biological Process (GOBP)(1000) refers to 1000 randomly sampled gene sets from GOBP. (C) Proportion of gene sets where the smallest overrepresentation *P*-value is associated with a specific model. (D) We identified 1418 gene set descriptions with cosine similarity >0.7 between the GOBP database and KEGG, Reactome, and WikiPathways. These gene sets allowed comparisons between human curators and established a baseline for human performance in gene set annotation. Gene sets were generated using LLMs based on KEGG, Reactome, and WikiPathways descriptions. (E) Fraction of gene sets significantly enriched in the GOBP reference across LLMs and human curators. “Human” refers to curator B from (D). (F and G) Boxplots showing recall and precision of gene sets generated by human curator B and LLMs.

To evaluate the output of an LLM, we assessed whether the gene sets generated by an LLM were significantly overrepresented in corresponding human-curated gene sets (Fig. 2A). To compute whether the overrepresentation we observed was not due to chance, we used the hypergeometric distribution to compute a *P*-value for a one-tailed Fisher’s exact test and adjusted for multiple testing (see Section 2). We performed this assessment using natural language descriptions of gene sets in three human-curated gene set databases: KEGG (Kanehisa and Goto 2000), Reactome (Joshi-Tope *et al.* 2005), WikiPathways (Martens *et al.* 2021), and 1000 randomly sampled gene sets from Gene Ontology Biological Process (GOBP) database (Ashburner *et al.* 2000). Curated for different applications, each gene set database captured differing underlying biology and varied in a total number of gene sets (Fig. S2A). In total, we examined overrepresentation in 3939 gene sets and the gene sets varied in size ranging from a median of 24–78 genes (Fig. S2B). We evaluated LLM gene set generations from three different language models: GPT-3.5, GPT-4o-mini, and GPT-4o (Brown *et al.* 2020, OpenAI *et al.* 2024). We found that between 33% and 94% LLM-generated gene sets were overrepresented in human-curated gene sets at a Bonferroni-adjusted *P*-value of .01 (Fig. 2B). We observed a higher fraction of overrepresented gene sets generated by the more capable GPT-4o model than GPT-3.5. This fraction tracked with the relative capabilities of each model we considered with GPT-4o mini ranking between GPT-4o and GPT-3.5. Overall, all models performed worst on the GOBP gene set database. Across most gene sets, the overrepresentation *P*-value was smallest for GPT-4o indicating that on an individual gene set level a more capable model yields gene sets in greater agreement with curated gene sets (Fig. 2C).

We next sought to compare how LLMs might perform head-to-head with human curators. To do so, we focused on 1418 gene sets in KEGG, Reactome, and WikiPathways (query gene sets) that had highly similar descriptions (cosine similarity >0.7 using a text embedding model, see Section 2) to the gene set descriptions in the entire GOBP gene set database (reference gene sets) (see Fig. 2D). Although they have highly similar descriptions, they originate from different databases and thus could be assumed to have been curated by different individuals. We found that 70% of these query gene sets from KEGG, Reactome, and WikiPathways were significantly enriched in the GOBP reference at a Bonferroni-adjusted *P*-value of .01 (Fig. 2E). GPT-4o performed similarly at this task, with 69% of gene sets enriched in the human GOBP reference. We also computed the precision and recall of genes across these gene sets for LLMs as well as humans (Fig. 2F and G). We found that GPT-4o generated gene sets more conservatively than human curators with lower recall (*P* < 10^−13^, Wilcoxon rank sum), but higher precision (*P* < 10^−12^, Wilcoxon rank sum).

The prompt we evaluated above relied on two additional components: a role-prompt and a format specification to generate outputs that can be used for downstream applications. Role prompting is a strategy by which to shape the output of an LLM by asking it to take on a specific role (see Section 2) (Schulhoff *et al.* 2024). We evaluated whether the role-prompt had any impact on the quality of the results. We found that the role-prompt had little impact on the evidence for overrepresentation of curated genes in LLM-generated gene sets (Fig. S2C). Formatting is crucial for subsequent use of the gene sets generated by LLMs. We assessed how often each LLM used HGNC symbols, as directed in the prompt, by comparison to gene symbols in Ensembl (GRCh38.p14, release 112). We found that on average 95% of the gene symbols returned by GPT-4o were HGNC gene symbols (Fig. S2D). This number was 88% for GPT-3.5 and GPT-4o mini. We additionally noticed a tendency of the models to return duplicate gene symbols for a given gene set description. We found that GPT-3.5 generated gene sets with one or more duplicate gene symbols across 13%–24% of gene sets, which was greatly reduced for GPT-4o (5%–6%) (Fig. S2E). Overall, the LLMs we evaluated followed formatting instructions enabling generation of HGNC gene symbols for downstream use.

Token use is a crucial metric for LLMs and directly tied to compute cost (Chen *et al.* 2023). Input tokens and output tokens are considered differently given that input tokens can be cached to reduce compute cost (Pope *et al.* 2022). We quantified the number of input and output tokens used to generate LLM version of each database and model (Fig. S2F). Given that GPT-4o and GPT-4o mini both use the same tokenizer, 707058 tokens were used as input to generate the gene sets using the descriptions from KEGG, Reactome, and WikiPathways. We found that GPT-4o used the most tokens in its output across databases. 1004497 output tokens were required to generate 3939 gene sets using GPT-4o. In contrast, GPT-4o mini required 756772 output tokens. Quantification of token usage indicates that the compute cost associated with gene set generation was minimal overall.

### 3.2 Ensembling and confidence improve the precision of gene set generation

**Figure 3.**
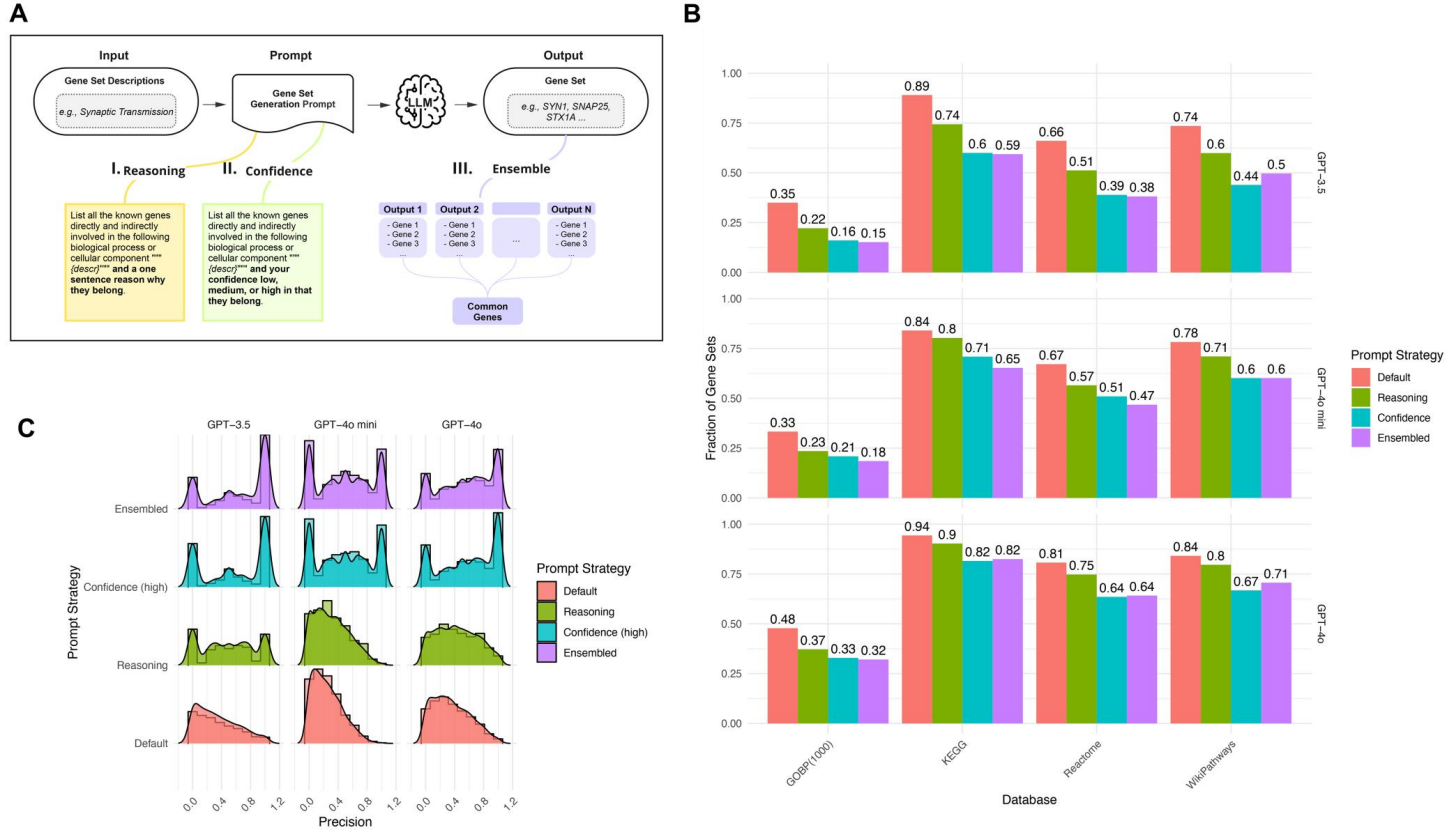
Assessment of model reasoning, confidence, and ensembling for gene set generation. (A) Strategies to generate gene sets using an LLM prompt. (B) Fraction of genes where the LLM-generated gene set is significantly overrepresented in the human-curated gene set with the same natural language description. Bar plot color designates one of three prompting strategies illustrated in (A). (C) Precision of the gene sets returned or the fraction of LLM-generated genes in a gene set that are in the human-curated gene sets with the same description.

LLM prompting is an active area of research (Schulhoff *et al.* 2024). On question-and-answer (Q&A) benchmarking datasets various prompting strategies have been explored to increase LLM performance. We evaluated three prompting strategies on the problem of gene set generation: model reasoning, model confidence, and ensembling (Fig. 3A, see Section 2). Encouraging an LLM to provide reasoning for why and how it arrived at a particular answer has been demonstrated to be a powerful approach to improving performance on Q&A datasets (Wei *et al.* 2024). We modified our prompt to require that the model provide a single sentence for its rationale for why a gene was included in a gene set (see Section 2). We additionally considered whether we could elicit the model’s confidence in whether it thought a gene belonged to a gene set. We required the model to indicate low, medium, and high confidence for each gene, limiting gene sets to high-confidence genes. We also considered ensembling. We used multiple generations of the model with differing random seed values. Across generations, we identified genes that the model consistently associated with a given gene set description. When a gene is consistently provided as output for a query for a gene set description, this may represent fixed and highly confident knowledge that the LLM captures.

For each of these approaches reasoning, confidence, and ensembling, we observed a small decrease in the number of LLM-generated gene sets that were overrepresented in human-curated gene sets (Fig. 3B). The impact was more modest for GPT-4o. As expected, all of the gene sets generated by ensembling were the same size or smaller than those generated by our original prompt. We hypothesized that genes that appeared consistently in model generations with different seeds reflected the model’s higher confidence that the gene belonged to a given gene set. To quantify this, we evaluated whether these gene sets were more precise. Precision can be quantified by the number of genes in the LLM-generated gene set that belong to a curated gene set over the total number of genes returned. We found that both ensembling and model confidence, but not model reasoning increased the number of gene sets with high precision (Fig. 3C). These gene sets were only present when gene sets were restricted solely to high-confidence genes, but not medium- or low-confidence genes (Fig. S3A). We examined the overlap between genes generated by ensembling and model confidence. Among gene sets that were significantly enriched in curated gene sets, we found a range of overlap between the gene sets generated by these approaches indicating that ensembling yields different genes than model confidence (Fig S3B). Each of the strategies we considered requires additional tokens, thus increasing cost. As expected, token usage was highest for the ensembling strategy followed by model reasoning (Fig. S3C and D). Taken together, our results illustrate that prompting strategies can tune the precision of gene sets generated by LLMs.

### 3.3 LLM-generated gene sets enable discovery of multiple enriched biological processes

LLMs have recently been proposed as tools to discover biological processes represented by an input set of genes (Hu *et al.* 2025). In this setting, an LLM prompt includes a set of genes and asks an LLM to generate a natural language description of the biological process these genes participate in—the reverse of our evaluation approach above. The prompt is evaluated based on how closely the LLM can recover the ground truth natural language description of the gene set in the output. In contrast to simple LLM prompting, *llm2geneset* generates a custom gene set database given a query gene set (Fig. 1). As LLMs may not use the same exact description as a curator used for a gene set, agreement can be quantified by, for example, how many words (unigrams) or word pairs (bigrams) from the ground truth description are present in the output of the LLM gene description. Agreement can also be quantified by the use of text embedding models and computation of cosine similarity between an embedding of the LLM description and an embedding of the ground truth description for a gene set (Cao 2024).

**Figure 4.**
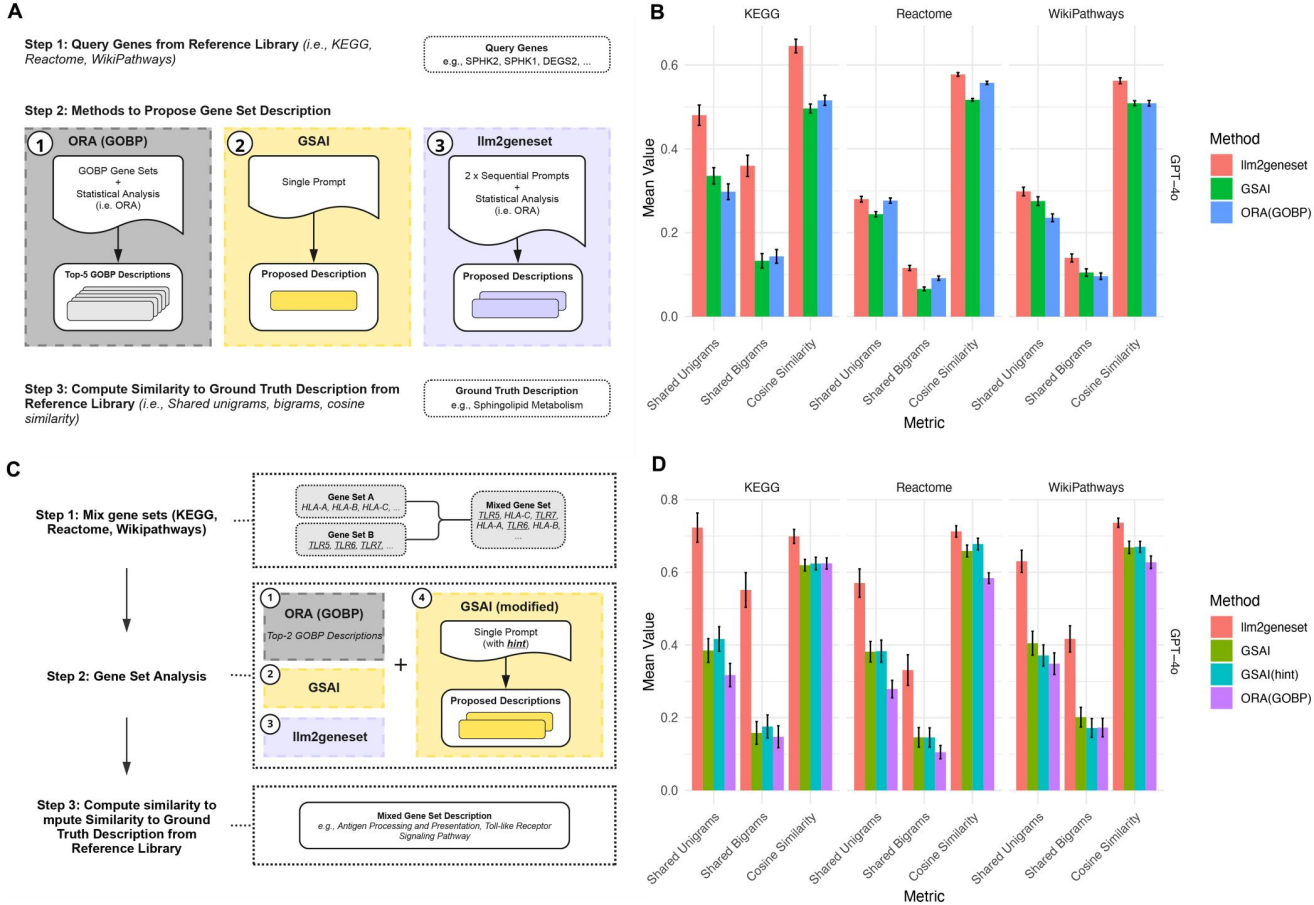
Gene set proposal enables discovery of multiple enriched biological functions. (A) Evaluation approach for traditional ORA using the GOBP database, GSAI, and *llm2geneset*. An input set of genes was provided. Each method was then allowed to propose gene set descriptions. These descriptions were compared using shared unigrams, bigrams, and cosine similarity with the ground truth description. (B) Mean fraction of shared unigrams (words) and bigrams (word pairs) and mean cosine similarity between gene set descriptions returned by the GSAI prompt and *llm2geneset* across databases for the GPT-4o model. Error bars designate the standard error of the mean. (C) To evaluate the ability of the approaches to identify multiple gene functions, we mixed gene sets that were easily identified by the approach in (A) and asked if each method recovered the mixed gene set descriptions. We also tested a modified GSAI prompt where we provided a hint indicating that two gene sets were present. (D) Mean fraction of shared unigrams (words) and bigrams (word pairs) and mean cosine similarity between gene set descriptions returned by GSAI, traditional ORA using GOBP, *llm2geneset* and the gene set descriptions originally assigned to mixed gene sets. Error bars designate the standard error of the mean.

We initially established a baseline using traditional ORA. To do so, we used query gene sets from KEGG, Reactome, and WikiPathways. We then used the GOBP database to identify (from GOBP) gene sets that were significantly enriched in the query gene sets (from KEGG, Reactome, and WikiPathways). We compared the “ground truth” descriptions of query gene sets (from KEGG, Reactome, and WikiPathways) to the top-5 significant gene sets returned by performing ORA using GOBP (Fig. 4A). Across these significant gene set descriptions from GOBP, we used the maximum shared unigrams, bigrams, and cosine similarity between the “ground truth” descriptions from the original query gene sets to assess the quality of the results.

We also compared our approach to an LLM prompting strategy called GSAI (Hu *et al.* 2025). In contrast to our approach, GSAI used a single prompt to request the LLM return the biological process the input genes belong to along with both reasoning and confidence. Using benchmarking gene sets from KEGG, WikiPathways, and Reactome, we found that *llm2geneset* generated gene set descriptions had on average a higher fraction of shared unigrams and bigrams as well as higher cosine similarity with the ground truth gene set descriptions than GSAI and outperformed the ORA baseline (Fig. 4B, Fig. S4A). This observation was highly significant for the GPT-4o model using shared bigrams (Wilcoxon rank sum, *P* < 10^−13^ for KEGG, *P* < 10^−5^ for Reactome, and *P* < 10^−5^ for WikiPathways) and cosine similarity (Wilcoxon rank sum, *P* < 10^−11^ for KEGG, *P* < 10^−17^ for Reactome, and *P* < 10^−9^ for WikiPathways) as evaluation metrics. In terms of computational cost, our approach required a comparable number of input tokens to GSAI, but a larger number of output tokens (Fig. S4B).

Experiments often influence multiple biological processes, leading to the discovery of distinct overrepresented gene sets that include DEGs. To simulate such a scenario, we selected gene sets where the biological processes were recovered by both the GSAI prompt and *llm2geneset* with high similarity to the ground truth gene set description (>0.7 cosine similarity) for each benchmarking gene set database. Next, we combined 50 pairs of these gene sets from each gene set database. We then evaluated the recovery of the distinct biological processes from these mixed gene sets, comparing the GSAI prompt with our approach across LLMs (see Section 2).

We established a baseline using traditional ORA by measuring text similarity between the concatenated text of the top 2 significant gene sets returned from the GOBP database. We found that with the GSAI prompt LLMs were frequently unable to identify multiple gene functions in a gene set. This also held when we modified the original GSAI prompt to include a text “hint” that indicated that two distinct gene sets were present in the input query gene set (see Section 2). Compared to both GSAI, GSAI with a two gene set hint, and the ORA baseline, *llm2geneset* more frequently recovered multiple functions as reflected by a higher fraction of shared unigrams and bigrams as well as cosine similarity (Fig. 4D, Fig. S4C). This observation was highly significant for the GPT-4o model using shared bigrams (Wilcoxon rank sum, *P* < 10^−11^ for KEGG, *P* < 10^−4^ for Reactome, and *P* < 10^−4^ for WikiPathways) and cosine similarity (Wilcoxon rank sum, *P* < 10^−3^ for KEGG, *P* < 10^−5^ for Reactome, and *P* < 10^−5^ for WikiPathways) as evaluation metrics. Our analysis indicates that coupling generation of gene sets by LLM to ORA is a more effective strategy than model prompting to uncover multiple distinct gene set functions within a set of genes.

### 3.4 LLM-based gene set generation enables steerable ORA

**Figure 5.**
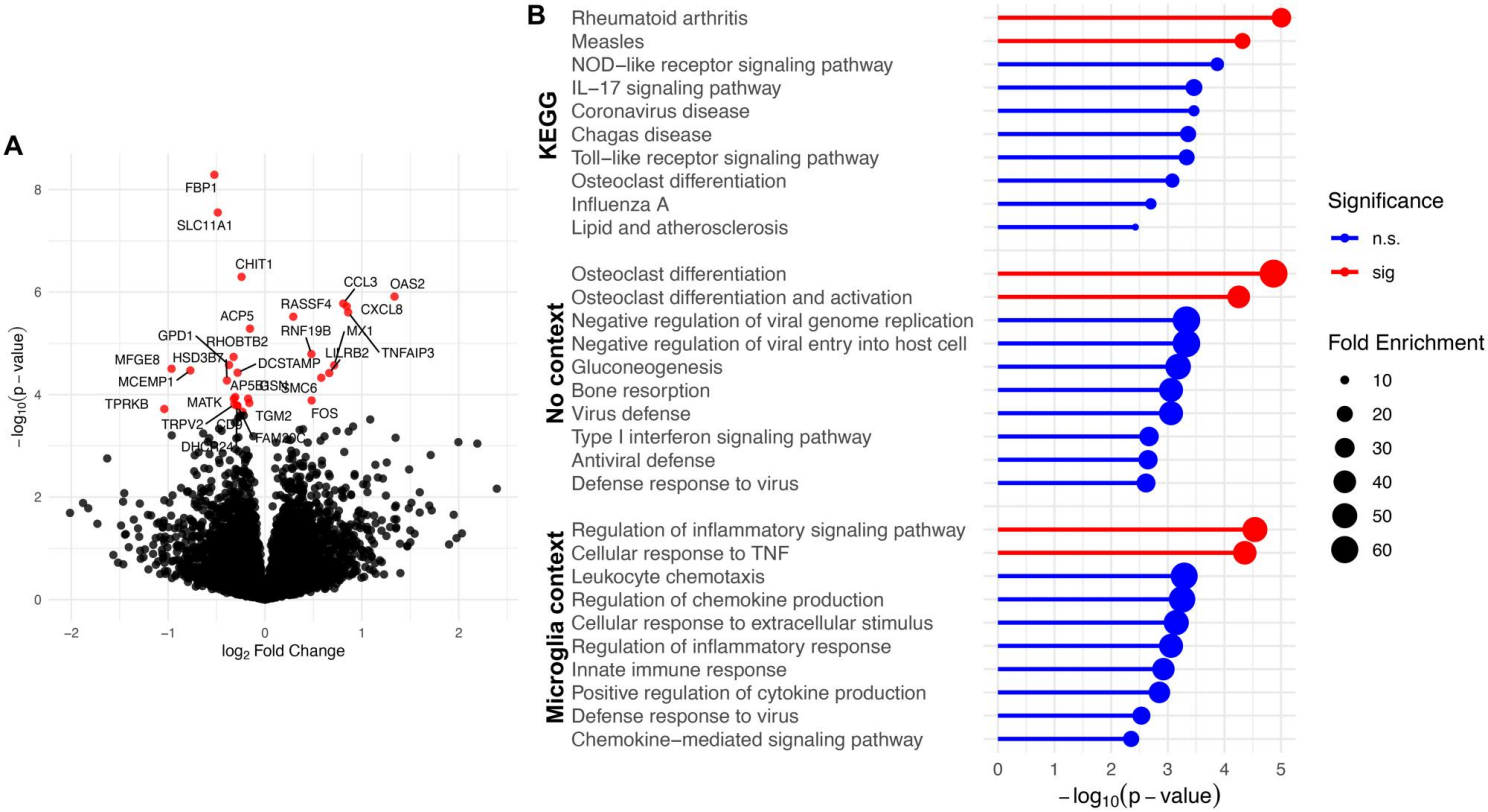
Comparison of overrepresentation analyses using human-curated (KEGG) and LLM-generated gene sets. (A) Volcano plot of DEGs comparing iPSC-derived microglia treated with a *TREM2* agonist antibody (AL002) to an IgG control antibody at 1ug/ml for 24 hours. Differentially expressed genes at FDR 10% are shown as red points. (B) Overrepresentation analysis results for biological process and pathways in: (top) the KEGG database; (middle) GPT-4o generated gene set database using no contextual information about the experiment; (bottom) GPT-4o generated gene set database using the contextual text “*in vitro* microglia treated with a *TREM2* agonist antibody.” The lollipop plot indicates the −log_10_(*P*-value) for overrepresentation of the gene sets in the set of DEGs in (a). Size of the circles in the plot designates fold enrichment of the gene set. Significant (sig) gene sets at an FDR of 1% are shown in red. Non-significant (n.s.) gene sets are shown in blue.

After establishing that our LLM-based approach is more effective than using a single prompt to identify multiple biological processes within a gene set, we applied this method to the DEGs from an experiment involving microglia differentiated from induced pluripotent stem cells (iMGs), treated with AL002 (*N* = 4) compared to an isotype control (*N* = 4) (see Section 2). AL002 is an investigational, humanized, *TREM2*-selective agonistic monoclonal antibody in Phase 2 trials for the treatment of early Alzheimer’s disease (Jackson *et al.* 2022). Activation of microglia is hypothesized to be an important mechanism of AL002. In iMGs, we found that 30 genes were differentially expressed at an FDR of 10% between iMG that received AL002 to the isotype control (Fig. 5A).

Given that it is difficult to define ground truth overrepresented gene sets from RNA-seq data, we qualitatively compared the overrepresentation results of using a fixed database derived from KEGG to that of results of *llm2geneset* (Fig. 5B). Using the KEGG database, we found several highly ranked pathways that were irrelevant to microglia. These include pathways of rheumatoid arthritis and measles which provide little information on the impact of AL002 *in vitro*. In contrast, a larger number of highly ranked pathways related to immune and osteoclast function were detected by *llm2geneset*. The enrichment in osteoclast function was driven by DEGs such as *ACP5* and *DCSTAMP*, which have also been associated with functions in the human immune system (Yagi *et al.* 2005, Briggs *et al.* 2011, Garcia-Hernandez *et al.* 2022). In this setting, the LLM was unaware of the experimental context of the input DEGs.

To address this scenario, we leveraged the ability of *llm2geneset* to steer the model toward gene set descriptions related to immune and microglial function (see Section 2, Fig. 5B). Here, the gene set proposal prompt included the context “*in vitro* microglia treated with a *TREM2* agonist antibody.” Using this approach we found the biological process “regulation of inflammatory responses” was significant at FDR 1%. This result is consistent with *TREM2*’s function of the innate immune system in the brain (Ulland and Colonna 2018). Pathways and biological processes related to immune function were ranked highly overall and gene sets related to osteoclast function were not tested. Overall, this example illustrates that LLMs can be steered to generate gene set databases highly relevant to a specific RNA-seq experiment. These gene sets can be evaluated for overrepresentation in a set of DEGs to facilitate the interpretation of RNA-seq datasets and subsequent hypothesis generation.

## 4 Discussion

Human-curated gene sets are difficult to tailor to specific input DEGs or proteins, as well as to the contextual details of the experiments that generated them. This limitation can lead to incorrect inferences about the biological processes impacted. *llm2geneset* offers an approach to dynamically generate gene set databases using LLMs, tailored to input genes and contextual information. It provides distinct advantages for gene set enrichment analysis by enabling researchers to customize gene sets based on the unique characteristics of their data and scientific questions, addressing a key limitation of human-curated databases. Additionally, *llm2geneset* leverages LLMs trained on continuously updated scientific literature, allowing researchers to update gene sets as scientific knowledge progresses. This provides a potential solution to the challenges associated with maintaining an up-to-date gene set database (Wadi *et al.* 2016). Furthermore, *llm2geneset* can be seamlessly integrated into existing gene set enrichment analysis methods, such as GSEA, Enrichr, and CAMERA. The generated databases can be readily used by these methods, enabling them to leverage the flexibility and adaptability of our framework while maintaining compatibility with established approaches. To broaden accessibility, a web application (https://llm2geneset.streamlit.app), enables researchers to use *llm2geneset* without programming expertise, expanding its utility for experimental biologists (Fig. S5).

A single prompt, given a set of input query genes such as DEGs, can be used to prompt language models to infer the function of the input genes (Hu *et al.* 2025). However, we find that this approach is less effective than *llm2geneset* at inferring the function of a given input set of genes, particularly when multiple gene sets are mixed. This advantage arises from our framework’s ability to propose and evaluate multiple candidate gene sets against the input query, enabling the discovery of distinct biological functions within complex gene sets. Additionally, a single prompt is more susceptible to being influenced by researchers steering the model too strongly through prompt phrasing. In contrast, steering within *llm2geneset* impacts only the proposed gene set descriptions, not the gene sets themselves, as the framework validates all generated gene sets against the input query genes.

Our work has several limitations. Certain gene sets cannot be recovered using LLM prompting alone. Integrating retrieval-augmented generation may enhance performance, but careful benchmarking is necessary to validate improvements (Gao *et al.* 2024). Our comparison between LLMs to human curators is limited to 1,418 gene sets. How human curators perform relative to LLMs needs further study. Our evaluation approach is knowledge-intensive, and one could argue primarily a test of the ability of LLMs to retrieve gene sets from their training data or pattern match against gene symbols and functions associated with genes. Evaluating whether reasoning approaches improve performance in inferring gene set function from DEGs given experimental context remains an area of future work (Hao *et al.* 2024). Additionally, our study evaluates only three LLMs—GPT-3.5, GPT-4o mini, and GPT-4o. Differences in model architectures, training approaches, and inference algorithms must be further examined using the gene set tasks and ORA baselines introduced in this work. Some gene sets are molecularly defined based on transcriptomics data, and our direct prompting approach may not recover these sets (Liberzon *et al.* 2015). However, LLMs’ ability to generate code could enable the automated creation of such gene sets, or models trained on expression data may address this limitation (Theodoris *et al.* 2023, Jiang *et al.* 2024). Despite these challenges, we anticipate LLMs will be essential tools for deriving insights from high-dimensional transcriptomics and proteomics datasets.

## Supplementary Material

vbaf054_Supplementary_Data

## Data Availability

All code, notebooks, and benchmarking datasets are available: https://github.com/Alector-BIO/llm2geneset. The prompts used in this study are available for download: https://github.com/Alector-BIO/llm2geneset/tree/main/src/llm2geneset/prompts. A streamlit web application incorporating this tool is available: https://github.com/Alector-BIO/llm2geneset/tree/main/webapp. An instance of the web application can also be accessed: https://llm2geneset.streamlit.app.
