# Supplementary material for: Enhancing gene set overrepresentation analysis with large language models
Source: Bioinform Adv. 2025 Mar 13;5(1):vbaf054. doi: 10.1093/bioadv/vbaf054 (PMC12093311; doi:10.1093/bioadv/vbaf054)
Supplement: vbaf054_Supplementary_Data [file vbaf054_supplementary_data.pdf]

Supplementary Methods

LLMs and Gene Sets Databases

We used the following versions of OpenAI large language models: GPT-4o mini (gpt-4o-mini-2024-07-18), GPT-3.5 (gpt-3.5-turbo-0125), and GPT-4o (gpt-4o-2024-08-06). GMT files containing gene sets were downloaded from https://maayanlab.cloud/Enrichr/#libraries. We used the Reactome (Reactome_2022), KEGG (KEGG_2021_Human), WikiPathways (WikiPathway_2023_Human), and the Gene Ontology Biological Process (GOBP, GO_Biological_Process_2023) gene set databases for evaluation. Each of the gene set descriptions were cleaned to remove any unique identifiers. To favor reproducible outputs from the LLMs, we used the seed parameter in the model API. We obtained the list of valid human HGNC symbols using the biomaRt R package by querying the `hsapiens_gene_ensembl` database for protein coding gene symbols (Durinck *et al.*, 2009).

Gene Set Generation

We used the following prompt to generate gene sets:

````
List all the known genes directly and indirectly involved in the following
biological process or cellular component """{descr}""". Use the following JSON
schema:
```json
{{
    "type": "array",
    "items": {{
        "type": "object",
        "properties": {{
            "gene": {{
                "type": "string",
            }}
        }},
        "required": ["gene"]
    }}
}}
```
The field `gene` is a gene involved in the following biological process or cellular
component: """{descr}""". Use the HUGO Gene Nomenclature Committee (HGNC) gene
abbreviations. Place the output in a JSON code block. Do not add any comments in
the JSON code block.
````

In the above prompt `{descr}` was replaced with a natural language description of a gene set. We used the following role prompt which we placed in the system message:

```
You are an expert in cellular and molecular biology.
```

The https://pypi.org/project/json-repair/ package was used to parse out the returned genes and repair any minor JSON formatting errors. If the JSON output was not repairable, we queried the model again with a different seed value. We used the input and output tokens returned by the model API, accumulating additional tokens if additional model queries were needed. We removed

any duplicate gene symbols returned by the LLM. The above prompt, query to an LLM, and parsing can be concisely written as a function that takes as input a string description of a gene set and returns a string list with the corresponding gene symbols:

```
G:List[str] <- GetGenes(D:str)
```

Evaluating Gene Sets Generated by LLM

We evaluated the quality of gene sets generated by the LLM by comparing them to human-curated gene sets with the same description. Specifically, we examined the overlap between LLM-generated gene sets and human-curated gene sets and used the hypergeometric distribution to perform a one-tailed Fisher's exact test to compute a p-value, assessing whether the observed overlap (or a higher overlap) could have occurred by chance. We used the hypergeom.sf function from the scipy.stats package to compute numerically accurate p-values. The total set of possible genes was assumed to be N = 19,846 human protein-coding genes, based on GRCh38.p14 statistics (https://useast.ensembl.org/Homo_sapiens/Info/Annotation). We corrected for multiple testing across gene sets using either Bonferroni correction or false discovery rate, as indicated. To evaluate model performance, we report the fraction of significantly overrepresented gene sets after multiple testing correction, which was applied across all gene sets within a database.

Comparison of LLM and Human Curator Performance

To evaluate the performance of large language models (LLMs) relative to human curators, we focused on 1,418 gene sets from KEGG, Reactome, and WikiPathways (query gene sets) with highly similar descriptions to gene sets in the GOBP database (reference gene sets). Similarity was determined using cosine similarity (>0.7) computed using OpenAI's text-embedding-3-large model. These query gene sets and reference gene sets were assumed to be independently curated as they originate from different databases. For each query gene set, we performed overrepresentation analysis (ORA) against the GOBP database, considering a Bonferroni-adjusted p-value threshold of 0.01 for significance. GPT-4o was evaluated using the same approach to generate gene sets for comparison. Additionally, precision and recall were computed for the genes included in the gene sets generated by LLMs and human curators. Statistical differences between LLMs and human curators were assessed using the Wilcoxon rank-sum test.

Additional Gene Set Generation Prompting Strategies

Prompts for confidence and reasoning closely followed our original prompt above. We modified the query portion of the original prompt to elicit model confidence as follows:

```
List all the known genes directly and indirectly involved in the following
biological process or cellular component """{descr}""" and your confidence low,
medium, or high in that they belong.
```

The formatting portion of the prompt included modifications to the output JSON schema to capture this additional confidence information. Only high confidence genes were used in our evaluation. Model reasoning was elicited in a similar manner with the query portion of the prompt modified as follows:

```
List all the known genes directly and indirectly involved in the following
biological process or cellular component """{descr}""" and a one sentence reason
why they belong.
```

Here, we also modified the JSON schema in the formatting portion of the prompt to output such that the single sentence corresponding to the model reason was captured. Ensembling was conducted by performing 5 generations of gene sets with different seed values for the model to obtain different genes for each generation. Genes that were consistently observed in all 5 generations were returned as the genes for the given gene set description.

Proposing Gene Set Descriptions from a Set of Genes

We used the following prompt to propose a set of gene set descriptions from a list of genes.

````
List {n_pathways} biological pathways, biological processes, or cellular components
that contain the following genes """{genes}""" with high confidence. Be as specific
as possible. List non-overlapping pathways, processes, or components. Do not
include the gene names in the outputs. Use the following JSON schema:
```json
{{
    "type": "array",
    "items": {{
        "type": "object",
        "properties": {{
            "p": {{
                "type": "string",
            }},
        }},
        "required": ["p"]
    }}
}}
```
Example output will look like the following:
```json
[{{"p":"BP or Pathway 1"}},
 {{"p":"BP or Pathway 2"}},
 {{"p":"BP or Pathway 3"}},
 {{"p":"BP or Pathway 4"}}
```
The element `p` designates a pathway, biological process or cellular component.
Place the output in a JSON code block. Do not add any comments in the JSON code
block.
````

In the above prompt, `{n_pathways}` was replaced with the number of desired biological pathways and process descriptions. `{genes}` was replaced by a comma separated list of genes for which we requested these biological processes and pathways. As with the gene set generation prompt, we used the https://pypi.org/project/json-repair/ package to parse out the returned genes and repair any minor JSON formatting errors. If the JSON output was not repairable, we queried the model again with a different seed value.

To steer the model toward experimentally relevant biological processes and pathways we additionally modified the above prompt to include contextual information when this contextual information was provided:

```
List {n_pathways} biological pathways, biological processes, or cellular components
that contain the following genes """{genes}""" with high confidence. Also consider
the following context as related to the genes: """{context}""" when selecting
pathways, processes, and components.
```

Here `{context}` was replaced with a user provided string that provided additional context to steer the model generations (e.g. "in vitro microglia treated with a TREM2 agonist antibody"). The above can be concisely written as a function that takes as input a list of genes D, a requested number of processes and pathways N, and optionally a C context string:

```
P:List[str] <- GetPathwaysProcesses(D:List[str], N:int, C:str)
```

Discovery of Multiple Enriched Biological Processes in Gene Sets

llm2geneset proposes pathways and biological process descriptions based on the input set of genes and an experimental context. These pathway descriptions, alone, are used to generate gene sets which are then tested for overrepresentation in the input set of DEGs. The `GetGenes()` function call below does not have access to any previous context. The parameters of the algorithm are as follows:

- D = set of DEGs (or any set of genes)
- N = number of biological pathways and processes to propose
- C = (optional) contextual information regarding the experiment from which the DEGs were obtained
- B = number of background gene sets for overrepresentation analysis

Pseudo-code is provided below:

```
llm2geneset(D:List[str], N:int, C:str)
      R = []
      P = GetPathwaysProcesses(D)
      for pathway in P:
            G = GetGenes(pathway)
            p = hgsf(|intersect(D,P)|- 1, B, |G|, |D|)
            R = R.append((pathway,p))
      return padjust(R)
```

In the above pseudo-code, the function `padjust()` computes q-values to account for multiple testing. `hgsf()` is one minus the cumulative distribution function (1-CDF) of the hypergeometric distribution. The function call computes the p-value according to a one-tailed Fisher's exact test. *llm2geneset* returns a list of N pathways sorted on their overrepresentation p-values.

GSAI Prompt Details

We used the previously published GSAI prompt (Hu *et al.*, 2025). The GSAI prompt provides extensive instructions to an LLM to provide a gene set description, a confidence value, and an analysis of how genes belong to the returned gene set description. We used regular expressions to parse out the gene set name, confidence, and the analysis. When we were unable to parse these outputs, we queried the LLM again with a different seed value to obtain outputs that could be parsed by regular expressions.

In our experiments with mixed gene sets below, we added the following "hint" to the GSAI prompt as the second line:

```
There are 2 distinct biological processes performed by this system of interacting
proteins.
```

Evaluating Biological Process Discovery from Gene Sets

We compared the performance of GSAI and *llm2geneset* through two experiments. In the first experiment, we used gene sets from KEGG, Reactome, and WikiPathways to evaluate how closely the outputs of the two methods matched the original gene set descriptions. The input gene sets were shuffled before being submitted to either GSAI or *llm2geneset*. We assessed similarity using three metrics: (1) the fraction of single words (unigrams) and (2) word pairs (bigrams) in the original descriptions that appeared in the returned descriptions, and (3) cosine similarity between text embeddings. Embeddings were computed using OpenAI's text-embedding-3-large model, and the dot product of the embeddings was used for comparison. The average similarity score across all gene sets was used to evaluate the two methods. For *llm2geneset*, which generates multiple gene sets (N=5), we only considered gene sets significant at a Bonferroni-corrected p=0.002. If no significant sets were found, we returned "None Found." The maximum score for each metric across the significant gene set descriptions was reported.

In the second experiment, we focused on gene sets where the cosine similarity between GSAI and *llm2geneset* descriptions was >0.7 in the first experiment. We randomly selected 50 such pairs, combined their genes, and shuffled the new gene sets. For GSAI, we used the parsed name from the LLM output, and for *llm2geneset*, we generated N=5 gene sets, again keeping only those significant at p=0.002. We combined the descriptions of the significant gene sets into a comma-separated string or returned "None Found" if no significant sets were identified. We compared the GSAI description and the *llm2geneset* description to a reference string, which combined the original human-curated descriptions in a comma-separated format. Similarity was quantified by the fraction of unigrams and bigrams from the reference string present in the GSAI and *llm2geneset* outputs, as well as the cosine similarity between the strings. The average similarity across all 50 combined gene sets was used to compare the two methods.

Bulk RNA-seq from iMGs Treated with AL002

Induced pluripotent stem cells (iPSCs) were differentiated into mature microglia as previously described (Haenseler *et al.*, 2017) . A total of 2.5 x 10^4 cells each well were seeded in a 96-well plate format, and after 7 days in culture, the cells were treated with AL002 or an isotype-IgG control at 1ug/ml and incubated for an additional 24h. Next, cells were harvested, and RNA was isolated using the RNeasy 96 QIAcube HT Kit (Qiagen, Hilden, Germany). The quality of RNA was assessed using the TapeStation System (Agilent Technologies, Santa Clara, CA). RNA samples

were converted to cDNA libraries using the Universal Plus mRNA-Seq kit with NuQuant (Tecan Genomics, Redwood City, CA). RNA sequencing was performed on an Illumina NovaSeq 6000 system using a NovaSeq V1.5 kit (SeqMatic, Fremont, CA), with 2x100 bp paired-end reads.

Sequencing quality was evaluated using FastQC (v0.12.1). Raw reads were trimmed and filtered using fastp (v0.23.4) to remove low quality bases and adapter sequences, discarding reads shorter than 25 bp(Chen *et al.*, 2018). Trimmed reads were aligned to the reference GRCh38 transcriptome with STAR (v2.7.11b)(Dobin *et al.*, 2013). Sequencing and alignment quality control was summarized with MultiQC (v1.19)(Ewels *et al.*, 2016). Differential expression analysis was conducted using DESeq2(Love *et al.*, 2014).

Supplementary Figures

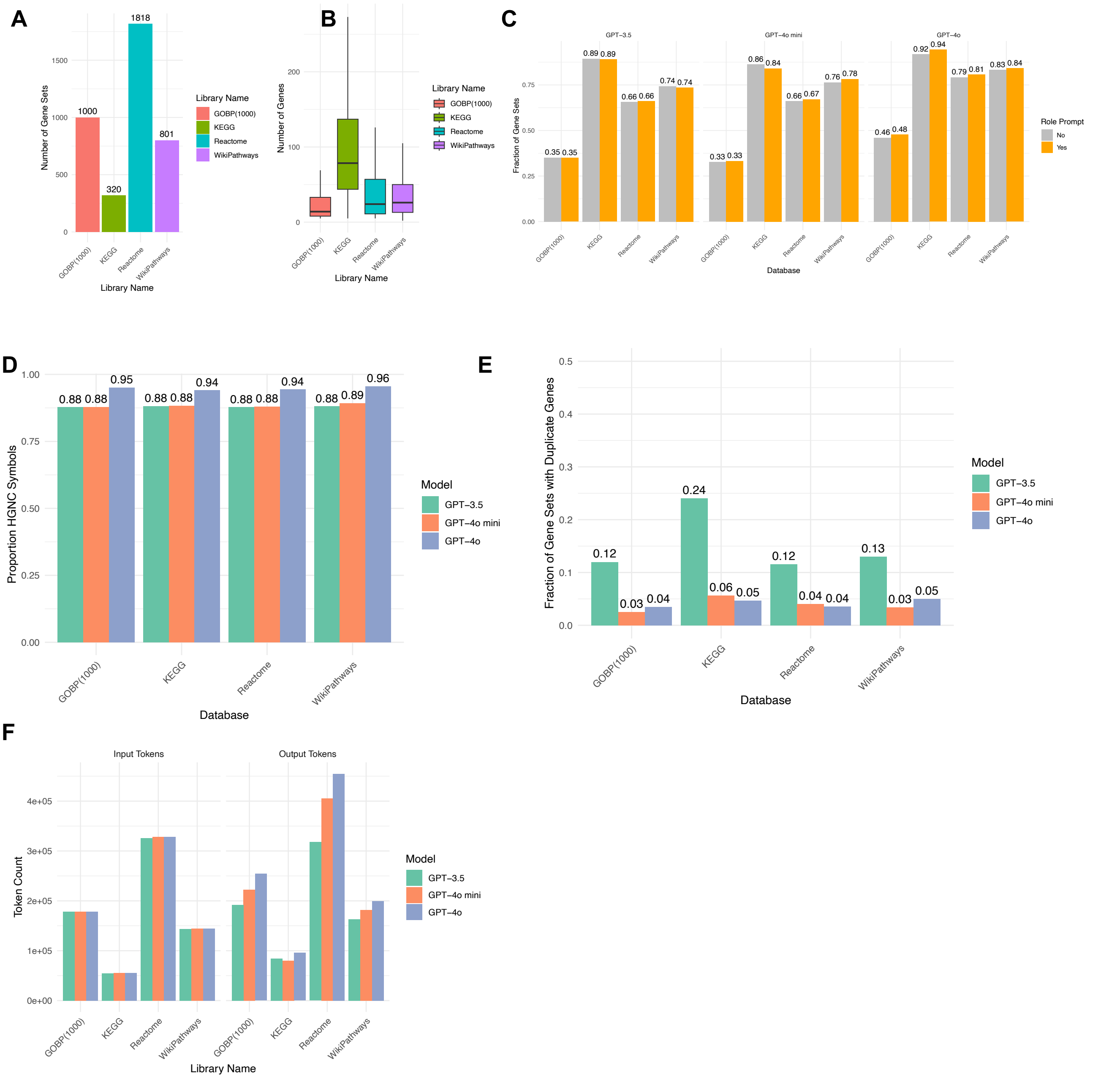

Figure S2. (a) Number of gene sets in the Reactome, WikiPathways and KEGG databases used for benchmarking. (b) Boxplot of the number of genes in each gene set across gene set databases. (c) Fraction of gene sets that show significant enrichment in human curated gene sets at a Bonferroni adjusted p-value of 0.01 across databases and LLMs. Plot compares the enrichment between gene sets generated using a role prompt and gene sets generated without a role prompt. (d) Fraction of gene symbols returned by LLMs across gene set databases that were known HGNC symbols in the Ensembl (GRCh38.p14) genome annotation. (e) Fraction of gene sets that contain duplicate genes across LLMs for each of the benchmark gene sets. (f) Total number of input and output tokens used to generate gene sets across models and databases.

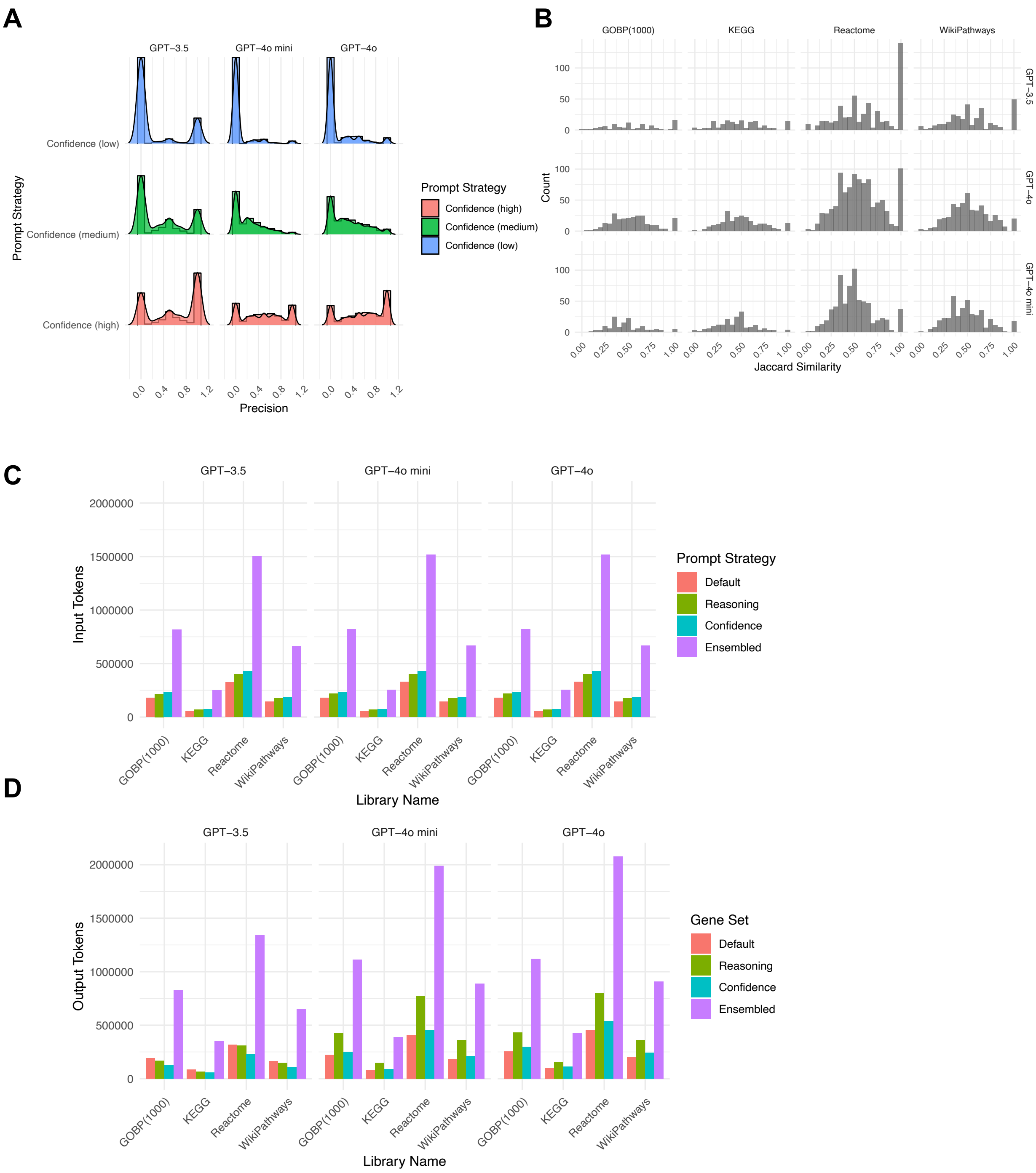

Figure S3. (a) Precision of gene sets comprising of only low, medium, and high confidence genes (b) Jaccard similarity between gene sets from the high confidence strategy and from ensembling that were significantly enriched in human curated gene sets at an adjusted p < 0.01 across databases and models. (c) Input tokens and (d) output tokens used by the default prompt strategy as compared to a prompting strategy that incorporates model reasoning, self-reported confidence, and ensembling.

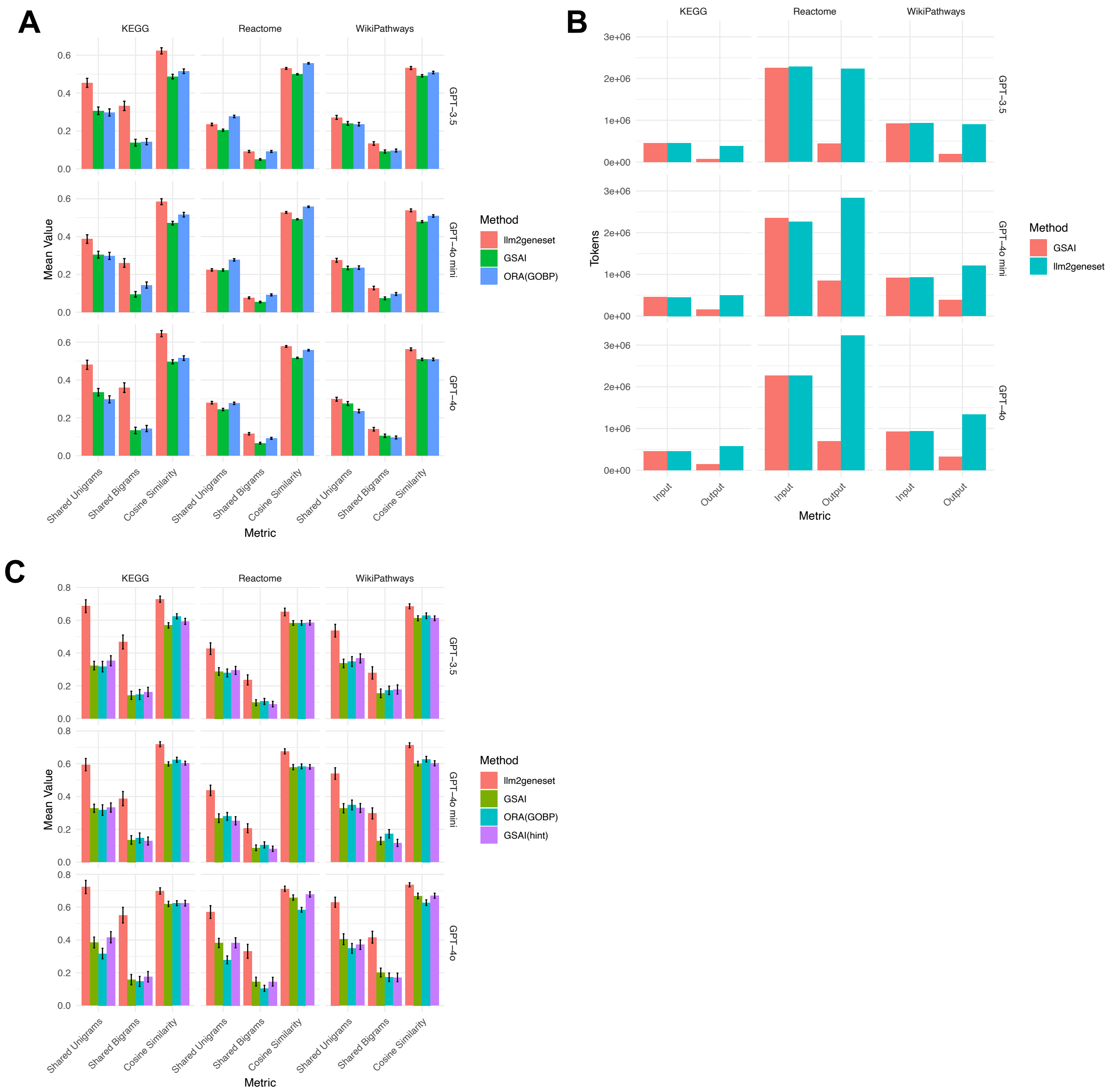

Figure S4. (a) Mean fraction of shared unigrams (words) and bigrams (word pairs) and mean cosine similarity between gene set descriptions returned by the GSAI prompt and *llm2geneset* across databases and LLMs. Error bars designate the standard error of the mean. (b) Input and output token usage of GSAI and *llm2geneset* across databases and models. (c) Mean fraction of shared unigrams (words) and bigrams (word pairs) and mean cosine similarity between gene set descriptions returned by GSAI and *llm2geneset* and the gene set descriptions originally assigned to mixed gene sets. Error bars designate the standard error of the mean.

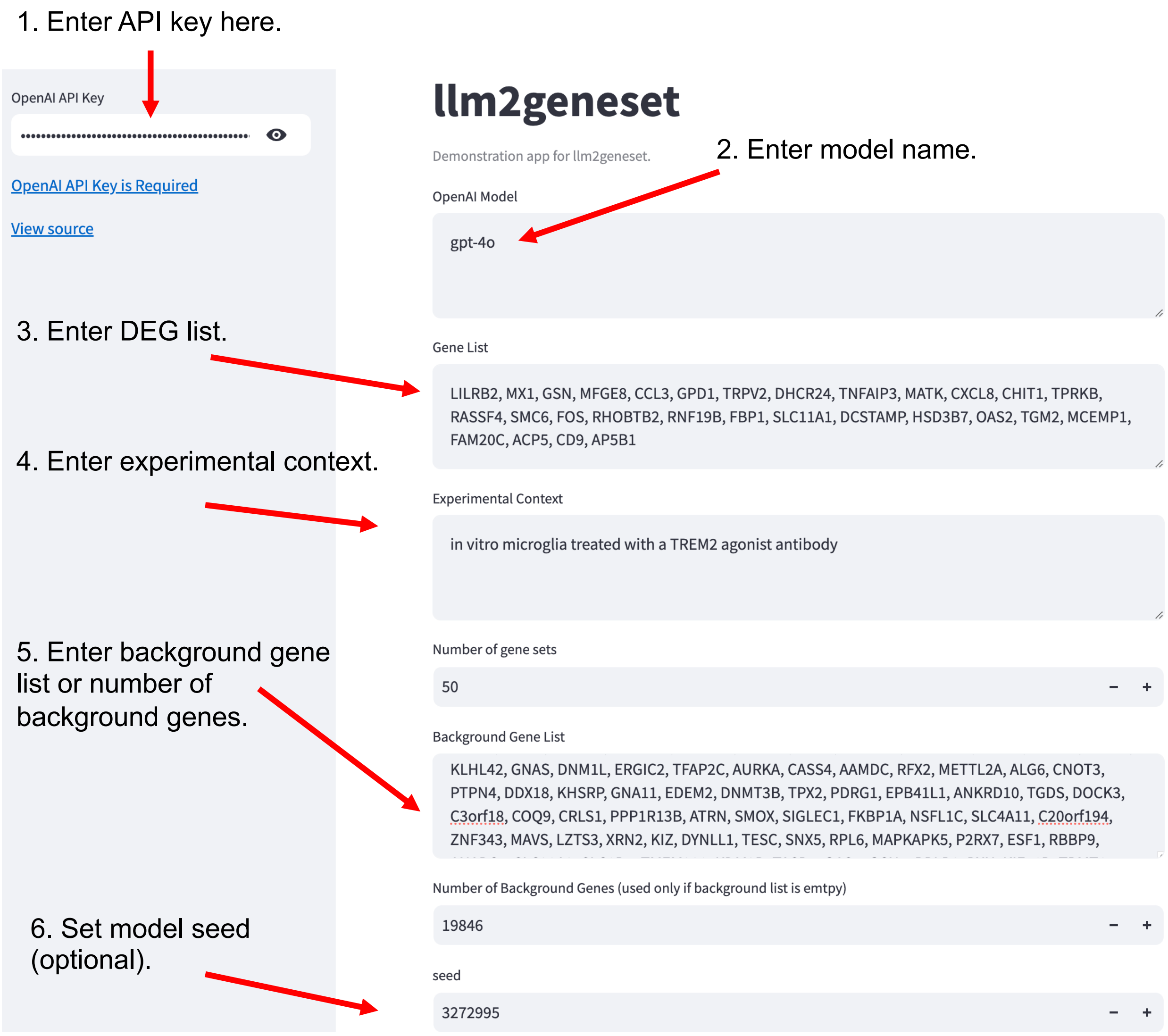

Figure S5. An illustration of how to use the demonstration application at https://llm2geneset.streamlit.app. To begin, the user must enter an API key and select a model. The input differentially expressed genes (DEGs) or proteins should be entered in the "Gene List" box. Additional experimental context can be provided in the "Experimental Context" box using natural language—this may include background information on the experiment or a specific question about the data. Users can also control the number of gene sets generated. Additionally, a background gene list can be provided, or the number of background genes can be set manually. The LLM seed parameter influences the variability of the model's outputs.

Supplementary References

Chen,S. *et al.* (2018) fastp: an ultra-fast all-in-one FASTQ preprocessor. *Bioinformatics*, **34**, i884–i890.

Dobin,A. *et al.* (2013) STAR: ultrafast universal RNA-seq aligner. *Bioinformatics*, **29**, 15–21.

Durinck,S. *et al.* (2009) Mapping identifiers for the integration of genomic datasets with the R/Bioconductor package biomaRt. *Nat Protoc*, **4**, 1184–1191.

Ewels,P. *et al.* (2016) MultiQC: summarize analysis results for multiple tools and samples in a single report. *Bioinformatics*, **32**, 3047–3048.

Haenseler,W. *et al.* (2017) A Highly Efficient Human Pluripotent Stem Cell Microglia Model Displays a Neuronal-Co-culture-Specific Expression Profile and Inflammatory Response. *Stem Cell Reports*, **8**, 1727–1742.

Hu,M. *et al.* (2025) Evaluation of large language models for discovery of gene set function. *Nat Methods*, **22**, 82–91.

Love,M.I. *et al.* (2014) Moderated estimation of fold change and dispersion for RNA-seq data with DESeq2. *Genome Biol*, **15**, 550.
